# A hybrid Chinese word segmentation model for quality management-related texts based on transfer learning

**DOI:** 10.1371/journal.pone.0270154

**Published:** 2022-10-07

**Authors:** Peihan Wen, Linhan Feng, Tian Zhang

**Affiliations:** 1 School of Management Science and Real Estate, Chongqing University, Chongqing, P. R. China; 2 College of Mechanical and Vehicle Engineering, Chongqing University, Chongqing, P. R. China; Menoufia University, EGYPT

## Abstract

Text information mining is a key step to data-driven automatic/semi-automatic quality management (QM). For Chinese texts, a word segmentation algorithm is necessary for pre-processing since there are no explicit marks to define word boundaries. Because of intrinsic characteristics of QM-related texts, word segmentation algorithms for normal Chinese texts cannot be directly applied. Hence, based on the analysis of QM-related texts, we summarized six features, and proposed a hybrid Chinese word segmentation model by means of integrating transfer learning (TL), bidirectional long-short term memory (Bi-LSTM), multi-head attention (MA), and conditional random field (CRF) to construct the mTL-Bi-LSTM-MA-CRF model, considering insufficient samples of QM-related texts and excessive cutting of idioms. The mTL-Bi-LSTM-MA-CRF model is composed of two steps. Firstly, based on a word embedding space, the Bi-LSTM is introduced for context information learning, and the MA mechanism is selected to allocate attention among subspaces, and then the CRF is used to learn label sequence constraints. Secondly, a modified TL method is put forward for text feature extraction, adaptive layer weights learning, and loss function correction for selective learning. Experimental results show that the proposed model can achieve good word segmentation results with only a relatively small set of samples.

## Introduction

Advances in data analytics make data-driven automatic/semi-automatic quality management (QM) become possible, and it is a trend to incorporate different kinds of data analytics technologies to improve performance [1]. Deep learning is widely used in QM data analytics [2–5]. Current QM relevant research mainly focuses on numerical data-driven models, with little attention on text-based information mining, which has been fully utilized to improve management proficiency in knowledge management, risk management, customer management, *etc*. [6–9]. Therefore, it is worthy of introducing text mining to QM domain and making full use of latent information of a wide range of QM-related texts to improve QM proficiency and efficiency.

Chinese word segmentation (CWS) is the basic step for Chinese text mining because there are no obvious marks between characters to show the boundary of words [10]. Current CWS tools could be divided into three categories: rule-based methods, machine learning methods, and deep learning methods. Rule-based methods [11, 12] can efficiently match and segment words in a certain way by establishing a dictionary, but hardly ever deal with words out of rules. Machine learning methods such as Hidden Markov Model (HMM) [13], Perception [14], and Conditional Random Field (CRF) [15], transform Chinese texts to sequence labeling to achieve high word segmentation accuracy. Similarly, the essence of deep learning methods [16, 17] is also sequence labeling, which will be learned through multi-layer neural networks.

There are three main difficulties for CWS: word boundaries, ambiguous words, and unknown words [18]. Firstly, the boundaries of words are normally vague with no authoritative standards, so that word segmentation can be distinct due to the influence of miscellaneous factors. Secondly, due to the ambiguity of the meaning of a word, a sentence may be divided into several words in different ways. Thirdly, there may be words that did not occur during rule making or model learning.

In order to solve the above problems and enhance the learning ability of models, researchers proposed different kinds of methods. A recursive neural network (RNN) model was proposed to represent the input context by transmitting and extracting the information of the reset gate and the update gate [19]. Long short-term memory (LSTM) networks were put forth to solve the issue that neural networks use fixed windows to ignore long-range information [20]. The combination of LSTM and RNN makes it possible to consider both local features and long-range dependencies [21]. In addition, bidirectional LSTM (Bi-LSTM) networks can take full advantage of contextual information [22]. Since CRF is observed to be able to improve the performance of the traditional Bi-LSTM model, a Bi-LSTM-CRF model was proposed for sequence labeling tasks [23]. The word segmentation performance can be further improved by integrating an attention mechanism into the Bi-LSTM-CRF model [24]. Then a transfer learning mechanism was introduced to cope with the problem that a deliberately designed and trained model cannot work well on test data without similar application context and feature distribution to training data [25–28].

Nevertheless, the above methods cannot be directly applied to word segmentation for QM-related texts because of special characteristics as follows.

1. The territorial nature of QM-related words. QM-related words, such as "公称压力" (nominal pressure), "碳素钢" (carbon steel), etc., scarcely ever appear in normal texts, and lack enough labeled samples for training, leading to a high probability of biased patterns.
2. Regular grammatical combination rules of QM-related words. QM-related words may be composed in forms of “component + appearance/structure/size/other parameters”, or “material + component”, or other domain-specific rules, hence words are supposed to be segmented in accordance with the above rules. For example, words such as "钢管外径" (outer diameters of steel pipes), "表面粗糙度" (surface roughness), and "密封面直径" (diameters of sealing surfaces) obey the rule of "component + appearance/structure/dimension/other parameters". If a model can learn that "钢管" (steel pipes), "表面" (surfaces), and "密封面" (sealing surfaces) are components, whereas "外径" (outer diameter), "粗糙度" (roughness), and "直径" (diameter) are parameters, etc., then the model can segment out a word of "密封面直径" (diameter of sealing surfaces) according to a rule learned from labeled words such as "钢管外径" (outer diameters of steel pipes) and "表面粗糙度" (surface roughness).
3. Regular semantic combination rules of QM-related words. To represent a specific component, several restricted adjectives will be added to constrain a central word, which makes QM-related words longer and more complicated than words in normal texts. For example, in a word "大直径钢制管法兰" (large diameter steel pipe flanges), the central word “管法兰” (pipe flanges) is restricted with “大直径” (large diameter) in size and “钢制” (steel) in material. A word segmentation method for normal texts will divide the word into three parts as the size, the material, and the central word, respectively, i.e., “大直径” (large diameter), “钢制” (steel), and “管法兰” (pipe flanges), which fails to clearly express the original meaning.
4. Strong contextual relevance and high recurrence of characters. Quality parameters are not completely independent. Relationships between quality parameters enhance the relevance of QM-related texts, coupled with special grammatical combination rules, resulting in the repetition of some characters in different words. For example, a word "焊接" (welding) may appear alone or in words "焊接端" (welding ends), "焊接头" (welding head), etc., which leads to a long-distance dependency problem.
5. Various structural paradigms of QM-related documents. QM-related documents, including QM standards, product specifications, inspection reports, etc., have different structural paradigms. For example, QM standards mainly consist of texts, whereas inspection reports usually contain tables and diagrams besides texts. Texts extracted from tables are incomplete expressions, and texts extracted from diagrams may be randomly embedded in other sentences.
6. Incomplete expression of relationships between properties and corresponding values in form of tables. For example, two columns with "材料" (material) and "碳素钢" (carbon steel), respectively, of a table may mean "材料是碳素钢" (The material is carbon steel.), omitting a predicate “是” (is). Due to the missing of predicates, the feature distribution will be quite special compared with normal texts. Moreover, every subset of training data may contain incomplete expressions, resulting in repeated adjustments between special and normal feature distributions, which will eventually increase the difficulty of model learning.

**Fig 1:**
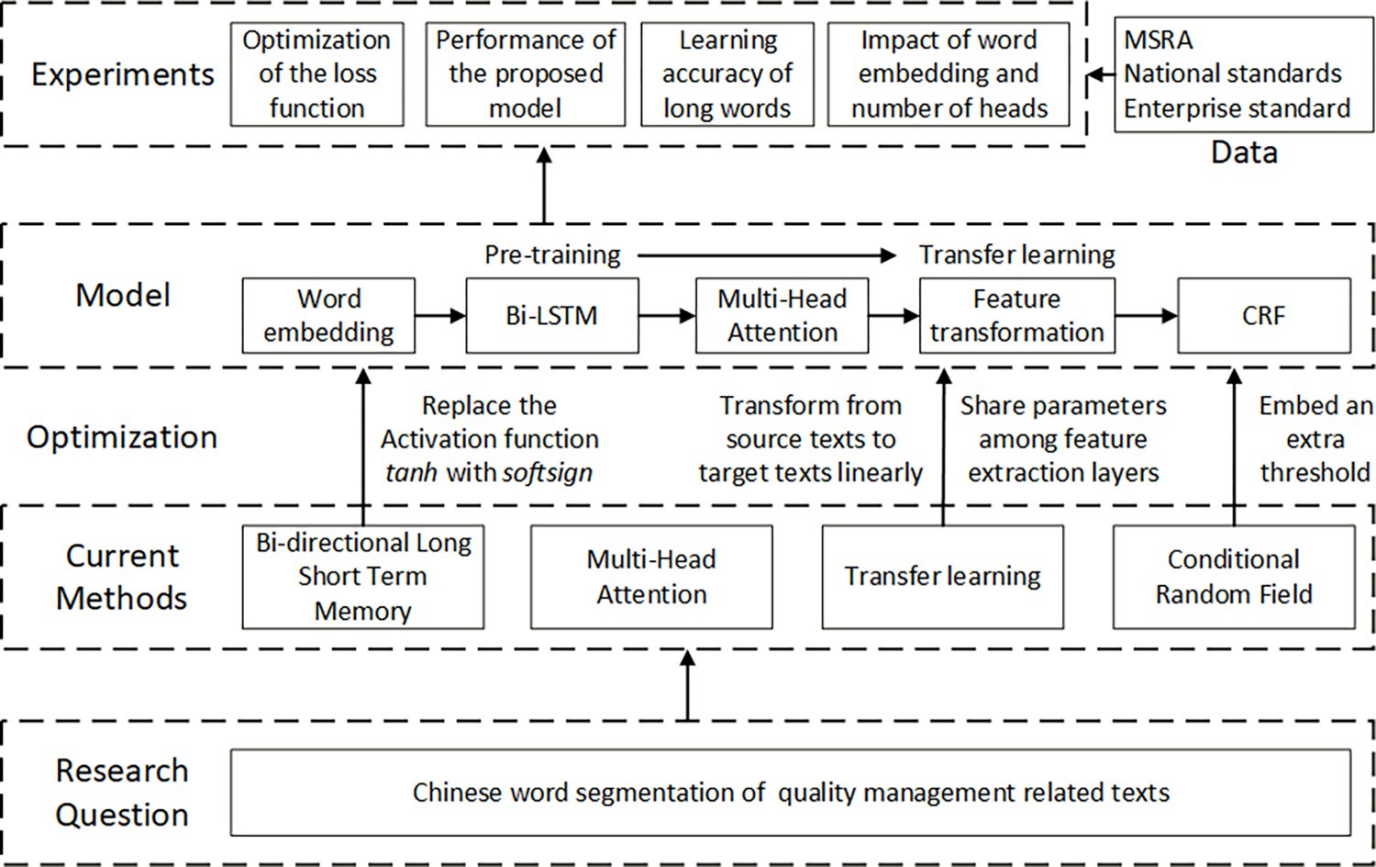
Methodological framework. A hybrid method and related CWS model of mTL-Bi-LSTM-MA-CRF are put forward for QM-related texts and validated with four sets of experiments.

The above analysis on characteristics of QM-related texts motivates us to develop a new CWS method specially for QM-related texts. We employ the concept of transfer learning (TL) to strengthen the learning ability of character features providing only a small set of samples, introduce multi-head attention (MA) to alleviate the long-distance dependency problem, and improve the loss function of CRF to focus on subsets with poor learning performance. Together with Bi-LSTM, a hybrid method is put forward to construct an mTL-Bi-LSTM-MA-CRF model, and validated with four sets of experiments on the loss function, learning accuracy of long words, segmentation performance, as well as impact of word embedding and number of heads, respectively, by means of QM standard documents of a key component of lots of complex products, the flange. The methodological framework is shown in Fig 1.

In summary, the contributions of this study are as follows.

1. A hybrid CWS model of mTL-Bi-LSTM-MA-CRF for QM-related texts is established by integrating transfer learning, multi-heads attention, Bi-LSTM and CRF. The proposed model has a better learning ability for long texts and alleviates problems of excessive segmentation and fewer training samples.
2. The optimization function of the loss function is designed and embedded with an extra threshold parameter to control the updating action. Consequently, the proposed model does not need to update related parameters if the loss value is lower than a predefined threshold, but only needs to selectively learn and update related parameters if the loss value is higher than the specified threshold, which improves the learning rate of the model.
3. The proposed model outperforms Jieba (a popular CWS tool for normal texts), the Bi-LSTM-CRF model, and the Bi-LSTM-CRF model with transfer learning.

### Preliminary

As methodological fundamentals, Bi-LSTM, multi-attention, and CRF, which will be further integrated and deliberately improved, are briefly introduced as follows.

### Bi-LSTM

The LSTM, which can remember long-distance dependency relationships within a period of time, solves the problem of feature loss existing in RNN when it encounters longer time steps [29, 30]. The LSTM consists of several systems, which are the input gate, the memory cell, the forget gate, the output gate, and the hidden state. The mathematical model of the LSTM is given by the following equations:

$$f_{t}=\sigma (W_{fx}x_{t}+W_{fh}h_{t-1}+b_{f})$$ (1)

$$i_{t}=\sigma (W_{ix}x_{t}+W_{ih}h_{t-1}+b_{i})$$ (2)

$$\tilde{C_{t}}=\tanh(W_{Cx}x_{t}+W_{Ch}h_{t-1}+b_{C})$$ (3)

$$C_{t}=f_{t}*C_{t-1}+i_{t}*\tilde{C_{t}}$$ (4)

$$o_{t}=\sigma (W_{ox}x_{t}+W_{oh}h_{t-1}+b_{o})$$ (5)

$$h_{t}=o_{t}*\tanh(C_{t})$$ (6)

Where *b*_*f*_, *b*_*i*_, *b*_*c*_ and *b*_*o*_ are bias of the forget gate, the memory gate, the cell state, and the input gate, respectively, $\tilde{C_{t}}$ is the temporary cell state, *C*_*t*−1_ is the cell state at the previous moment, and *h*_*t*_ is the hidden state. A *σ* is an activation function that determine to memory or forget information. A *tanh* activation function is applied to determine a temporary cell state $\tilde{C_{t}}$ and a hidden state *h*_*t*_, which can be replaced.

The LSTM encodes inputs with only consideration of the influences of previous texts on the current moment, whereas the Bi-LSTM considers context relationships fully [31, 32]. The Bi-LSTM is made up of two LSTM layers, considers influences of past and future information on the current moment, and encodes information between adjacent words in contexts. Therefore, the Bi-LSTM is used for feature extraction and encoding instead of the LSTM. For example, a backward hidden layer is marked as $\vec{h_{t}}=\{h_{l0},h_{l1},h_{l2},h_{l3}\}$, and a forward hidden layer is marked as $\overset{\leftarrow }{h_{t}}=\{h_{r0},h_{r1},h_{r2},h_{r3}\}$, then the output $h_{t}=\{[h_{l0},h_{r3}],[h_{l1},h_{r2}],[h_{l2},h_{r1}],[h_{l3},h_{r0}]\}$ is obtained by splicing the backward hidden layer state and the forward hidden layer state, which is denoted as $h_{t}=\{\vec{h_{t}},\overset{\leftarrow }{h_{t}}\}$.

### Multi-attention

The attention ignores distance relationships and gets degrees of interdependence between word vectors. A problem may arise that a model excessively focuses on its own position, so the multi-attention is proposed to solve the problem. The multi-attention encodes and extracts features from different subspaces [33, 34], which can learn internal structures of sentences better and faster and enhance expression ability.

Denote the input as *X* = [*x*_1_, *x*_2_, *x*_3_,…,*x*_*n*_], where *x*_*n*_ represents a vector of a single word, and *Q*, *K*, *V* are the query vector sequence, the key vector sequence and the value vector sequence, respectively. A general multi-attention model is formulated as:

$$Q_{i}=Q{W_{i}}^{Q},K_{i}=K{W_{i}}^{K},V_{i}=V{W_{i}}^{V}$$ (7)

$$head_{i}=attention(Q_{i},K_{i},V_{i})$$ (8)

$$multihead(Q,K,V)=Concat(head_{1},\ldots ,head_{h})W^{o}$$ (9)

Where *W*_*i*_^*Q*^, *W*_*i*_^*K*^ and *W*_*i*_^*V*^ represent different weight matrixes for *Q*, *K* and *V*.

The attention of a single attention head is given by the following equations:

$$attention(Q_{i},K_{i},V_{i})=\sum_{i=1}^{n}x_{i}\alpha_{i}$$ (10)

$$\alpha_{i}=soft\max(s(x_{i},Q))$$ (11)

### Conditional Random Field (CRF)

The CRF is a probabilistic undirected graph model that solves the conditional probability *p*(*y*|*x*) for a given input random variable *x* [35, 36]. A model uses the CRF with a linear chain to output the optimal CWS labeling sequence. Define a feature function *f*_*k*_(*y*_*t*−1_, *y*_*t*_, *x*_*t*_), *K* and *V* as state features, *y*_*t*−1_ as a transition feature, *Z*(*x*) is a normalization function, then the CRF model is formulated as:

$$p(y|x)=\frac{1}{Z(x)}\prod_{t=1}^{T}\exp\{\sum_{k=1}^{K}w_{k}f_{k}(y_{t-1},y_{t},x_{t})\}$$ (12)

## Methods

To segment Chinese words in QM-related texts, we integrate neural networks with the Bi-LSTM, the multi-attention, and the CRF to construct an mTL-Bi-LSTM-MA-CRF model.

### The mTL-Bi-LSTM-MA-CRF model

**Fig 2:**
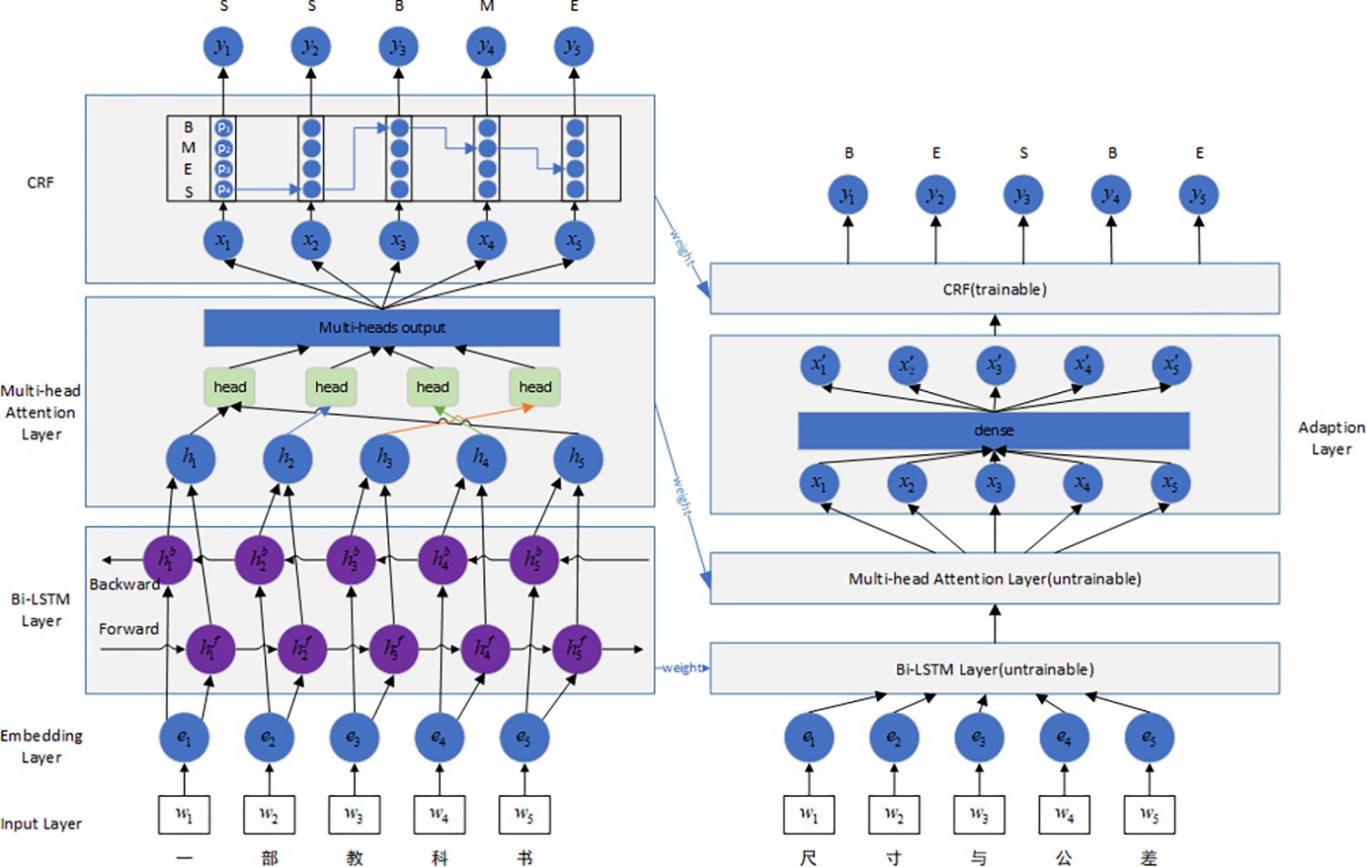
mTL-Bi-LSTM-MA-CRF model. Weights of the embedding, the Bi-LSTM, the multi-attention, and the CRF are pre-trained and shared with transfer learning. A weight of the adaptation layer is individually trained and a weight of the CRF is fine-tuned.

The mTL-Bi-LSTM-MA-CRF model contains two phases, one is pre-training, and the other is transfer learning, as shown in Fig 2. Our main improvements while constructing the proposed model include four parts, two for each phase, respectively. During pre-training, 1) the *tanh* function, which is always used for the activation function in the Bi-LSTM, is compared with the *softsign* function, leading to a replacement of activation function; and 2) the scaled dot-product function is selected by analysing calculation characteristics of several functions for scoring attention and the calculation requirements of the multi-attention. During transfer learning, 3) referring to the feature-based and model-based transfer learning, the proposed model shares the layers extracting features and learns the ability to transform features; and 4) the loss function of training is optimized by embedding an extra threshold parameter.

The implementation process of the proposed model is composed of seven steps in total, four steps for the pre-training phase, and three steps for the transfer learning phase.

#### The pre-training phase

General sequence labelling models [37–39] encode features of words by the embedding, extract the context relationships by the Bi-LSTM, and learn constraints of labeling sequence by the CRF. Therefore, a Bi-LSTM-MA-CRF model is pre-trained in a general domain.

*Vector embedding of words*. The embedding implements a transformation of inputs into vectors. General methods to represent words include the one-hot encoding and the word embedding [40]. The one-hot encoding cannot express relationships between words, and is too sparse to reduce the efficiency of calculation and storage, which is unsuitable for encoding texts. Whereas the word embedding changes dimension of words vectors from integer to floating point to compresses the original dimension, which may be used for texts representation. The word embedding expresses each character of inputs *x* = [*c*_1_, *c*_2_, *c*_3_,…,*c*_*m*_] as a feature vector to get vector matrixes of inputs *X* = [*C*_1_, *C*_2_, *C*_3_,…,*C*_*m*_]. Where the *c*_*i*_ represents the character of inputs, the *C*_*i*_ represents the feature vectors of the character *c*_*i*_, and the *m* is the length of inputs.

*Learning of context relationships*. The Bi-LSTM extracts context features of current inputs. A LSTM encodes the feature vector matrixes from front to back, whereas another one encodes from back to front. The output of the two LSTM are spliced in the dimension of word feature to get the output $h_{t}=\{\vec{h_{t}},\overset{\leftarrow }{h_{t}}\}$ of the Bi-LSTM.

*Learning of global information*. The recurrence of characters results in a requirement of learning of long-distance texts to segment accurately. But the information needs to be captured step by step in the Bi-LSTM, and the memory information slowly decays as the step size increases, leading to the important information may be discarded. Hence the multi-attention is introduced to allocate attention, weigh inputs, and extract important features autonomously, which enhances the learning of global information.

*Learning of sequence constraints*. The CRF can learn the constraints of the labelling sequence automatically to ensure the accuracy of final prediction results. Possible constraints are as follows: the beginning of sentences should be "B" or "S" instead of "M" or "E", the next labelling of "B" can only be "M" or "E", the next labelling of "M" can only be "M" or "E", and the ending of sentences should be "E" or "S" instead of "B" or "M". With the constraints, the error prediction of sequences will be greatly reduced.

#### The transfer learning phase

The pre-trained Bi-LSTM-MA-CRF model is trained in QM domain with a extra adaption layer.

*Extraction of feature*. The model extracts feature of QM-related texts with shared layers of the Bi-LSTM-MA-CRF model, including the features of words, the features of context relationships, the features of important global information.

*Learning of rules for feature transformation*. An adaptation layer learns transformation rules of the general and QM domain.

*Learning of sequence constraints again*. Considering the different rules of semantic combination between the source domain and the QM domain, constraints learned from the source domain may be improper when it used in the QM domain. So, the CRF has to be trained again to learn special constraints.

Next, we will specify the improvements of the replacement of activation function, the selection of function for scoring attention, the specific process of transfer learning, and the optimizing of loss function in the mTL-Bi-LSTM-MA-CRF model.

#### Replacement of activation function in Bi-LSTM

The activation function of the output gate can determine the temporary cell state $\tilde{C_{t}}$ and the hidden state *h*_*t*_ in the Bi-LSTM, where the *tanh* is used. And other activation functions for deep learning can also be used, such as *sigmoid*, *ReLU*, *softsign*, etc. The mathematical formulas of *softsign* and its derivative are as following:

$$f(x)=\frac{x}{1+|x|}$$ (13)

$$f^{\prime }(x)=\frac{1}{(1+|x|{)}^{2}}$$ (14)

**Fig 3:**
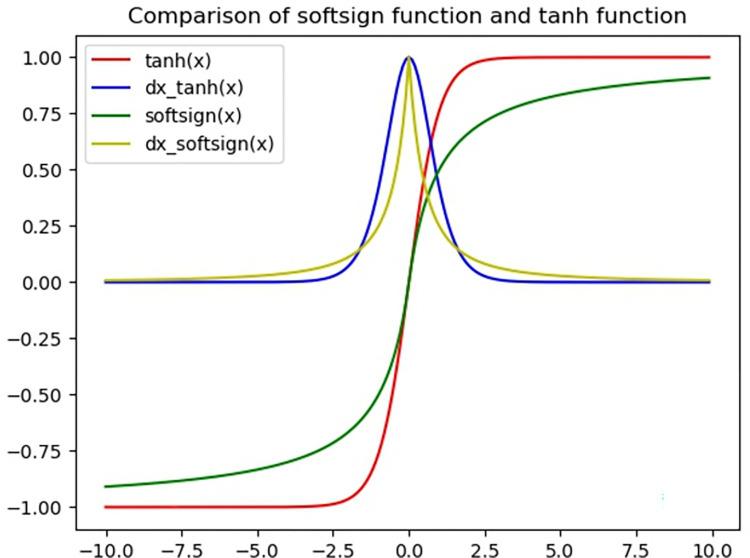
Comparison of softsign function and tanh function. A red line represents *tanh*, a blue line represents derivative of *tanh*, a green line represents *softsign*, and a yellow line represents derivative of *softsign*.

Fig 3 is a comparison diagram of *softsign* and *tanh*, where a red line represents *tanh*, a blue line represents derivative of *tanh*, a green line represents *softsign*, and a yellow line represents derivative of *softsign*. The curves of *softsign* and *tanh* are similar, and derivative curves are similar, too. But the curve of *softsign* is smoother than *tanh*, and it is less likely to reach saturation states. At the same time, the derivative curves of the two indicate slower downward trend of *softsign* derivative, so *softsign* is better at alleviating the phenomenon of gradient disappearance. Therefore, Eqs (3) and (6) are replaced by Eqs (15) and (16).


$$\tilde{C_{t}}=softsign(W_{Cx}x_{t}+W_{Ch}h_{t-1}+b_{C})$$ (15)

$$h_{t}=o_{t}*softsign(C_{t})$$ (16)

#### Selection of function for scoring attention

*s* (*x*_*i*_, *Q*) is a function for scoring attention, and common ones are the *additive* function, the d*ot-product* function, the *scaled dot-product* function and the *bilinear* function. In matrixes, product is more convenient than addition, so the *dot-product* function is better than the *additive* function. When the embedding dimension is small, the difference between the *dot-product* function and the *scaled dot-product* function is small, and the calculation rate of the *dot-product* function is higher than the *scaled dot-product* function. In the model, higher embedding dimensions may be better to represent textual features. Hence, the *dot-product* function has a larger variance than the *scaled dot-product* function, and the gradient of the *softmax* function will be smaller. The *scaled dot-product* function is more conducive to train model through a scaling factor $\sqrt{d_{k}}$. Therefore, the *scaled dot-product* function is selected to score attention:

$$attention(K,Q,V)=\sum_{i=1}^{n}x_{i}\alpha_{i}=soft\max(\frac{K^{T}Q}{\sqrt{d_{k}}})V$$ (17)

#### Transfer learning

For the characteristics of QM-related texts, learning of the character feature may give more support to segment words which is obey rules of grammatical combination, and transfer learning provides more opportunities for learning of the character feature, which means that transfer learning may improve the accuracy of CWS. In addition, labelling QM-related texts for training is insufficient. So, the transfer learning method is integrated into the model. A feature-based transfer learning reduces gaps between source domain and target domain by means of feature transformation. A model-based transfer learning finds parameters shared between the source domain and the target domain. A deep neural network is a module to extract feature intelligently, which captures complex nonlinear representations through multiple hidden layers, and all layers except the last layer usually are regarded as feature extractors [41]. Therefore, the proposed model combines the model-based and the feature-based transfer learning, extracting textual features through shared parameters, and transforming features by learning conversion rules.

*Sharing layers*. In the proposed model, the process from decoding by the word embedding to output by the CRF may be considered as a process of extracting from normal to special features of the training data. Large scale labelling data are used to pre-train weights of layers, and the weights actually reflect knowledges of extracting feature learned from training data. In transfer learning with QM-related texts, the model extracts character features *C*_*i*_ as well as contextual information with the weight of *W*_*BiLSTM*_, assigns appropriate attentions with the weight of *W*_*att*_ to obtain textual features *X*_*i*_ of inputs, and feeds the features to an adaptation layer composed of a fully connected network for transforming feature to realize the adaptability of QM-related texts.

*Adaption layer*. The model extracts feature parameters *x*_*i*_ of QM-related texts based on pre-trained knowledge by sharing parameters. However, it is a gap of textual features between the source domain and the QM domain, in terms of both language structures and character features. Therefore, it is need to learn rules of transforming feature to enhance the ability of extracting feature, which can be expressed as:

$${x_{i}}^{\prime }={activation(x}_{i}\cdot w_{trans})+bias$$ (18)

Assume that a rule of transforming textual feature between the source domain and the QM domain obeys a linear mapping without bias, so *linear* function may be used as activation function for the adaption layer, and Eq (16) is replaced by Eq (17):

$${x_{i}}^{\prime }=x_{i}\cdot w_{trans}$$ (19)

#### Optimizing of loss function

Losses shows differences between real values and predicted values, and are measured by a loss function. The smaller the losses are, the better the performance is. A mini-batch gradient descent is adopted to reduce losses continuously along the direction of a negative gradient of current position. Unless the loss function is a convex function, the direction of gradient descent may also be along a locally optimal instead of a globally optimal, that is, updates may be always invalid. In addition, considering an adjusting of model between incomplete expressions and normal texts, more attentions should be paid to the situation with a larger loss instead of a small loss which is caused by the different expression in QM-related texts. However, the model is updated continuous unless the loss equal to 0 during training. The loss is almost impossible to achieve to 0 in fact, so the model will be constantly updated. A model with *hinge* loss function updates parameters selectively and focuses on fuzzy forecast values. Referring to the function, an optimization function *ω*(Loss_*CRF*_, *n*) based on *sigmoid* function is designed to learn and update selectively by embedding a threshold.

The loss function of the CRF is generally used as a loss function of training which utilize the CRF to predict outputs. Two types of features in the CRF are state features and transition features. State features are represented by the score of the current node coming from the output of previous layer, whereas transition features are represented by the score from a previous node to a current node, and transition features are the weights of the CRF. A probable formula of correct path is the proportion of a true path in all possible paths as follows:

$${Prob}_{real}=\frac{P_{real}}{P_{1}+P_{2}+\cdots +P_{N}}$$ (20)

Therefore, the loss function of CRF is as follows:

$${Loss}_{CRF}=-(\sum_{i=1}^{N}x_{i,y_{i}}+\sum_{i=1}^{N-1}t_{y_{i},y_{i+1}}-log(e^{s_{1}}+e^{s_{2}}+\cdots +e^{s_{n}}))$$ (21)

The *Loss*_*CRF*_ represents the difference between score of total paths and real path. If score of total paths is equal to score of real paths, the loss is equal to 0. Setting a threshold as n, an optimization function *ω*(Loss_*CRF*_, *n*) is designed. *ω*(Loss_*CRF*_, *n*) tends to 1 when Loss_*CRF*_>*n*, whereas tends to or equal to 0 when Loss_*CRF*_≤*n*.


$${Loss}_{new}=\omega ({Loss}_{CRF},n){Loss}_{CRF}$$ (22)

According to the property, the *unit step* function (*x* > 0, *y* = 1; *x* = 0, *y* = ½; *x* <0, *y* = 0) may be considered. But the derivative is equal to 0 of *unit step* function in most situation, and the changes of outputs caused by small changes in parameters is directly erased, which is unconducive to update parameters. The limit value of *sigmoid* function (*x*→+∞, σ(*x*) = 1; *x* = 0, σ(*x*) = 1/2; *x*→−∞, σ(*x*) = 0) is consistent with *unit step* function, and *sigmoid* function is differentiable. Therefore, by entering an infinity parameter *m* in Eq (23), the *ε*(*x*)(Eq1 (24)) is obtained. The *ε*(*x*) (*x* > 0, *ε*(*x*)→1; *x* = 0, *ε*(*x*) = 1/2; *x* <0, *ε*(*x*)→0) is differentiable, and the derivative is not always equal to 0. However, the *ε*(*x*) is not equal to 0 when *x* = 0, so the *ω*(*Loss*_*CRF*_,*n*) should perform basic operations on *ε*(*x*), without changing property of derivability and value of positive or negative, to get a required value. The specific calculation is Eq (25).


$$\sigma (x)=\frac{1}{1+e^{-x}}$$ (23)

$$\varepsilon (x)=\underset{m\to \infty }{\lim}\left(\frac{1}{1+e^{-mx}}\right)$$ (24)

$$\omega ({Loss}_{CRF},n)=\varepsilon ({Loss}_{CRF}-n)-2\varepsilon ({Loss}_{CRF}-n)*\varepsilon (n-{Loss}_{CRF})$$ (25)

When Loss_*CRF*_>*n*, the *ε*(Loss_*CRF*_−*n*) approaches 1 infinitely, the *ε*(*n*−Loss_*CRF*_) approaches 0 infinitely, so the optimization function approaches 1 infinitely, and the optimization function does not interfere with the calculation of the original loss function. When Loss_*CRF*_ = *n*, *ε*(Loss_*CRF*_−*n*) = *ε*(*n*−Loss_*CRF*_) = 1/2, the optimization function is equal to 0; When Loss_*CRF*_<*n*,as *ε*(Loss_*CRF*_−*n*) goes to 0, the optimization function goes to 0. The value of loss function approaches 0 when optimization function approaches 0, and the model does not update.

Rules of update parameters are changed. *n* is a path score threshold. When the value of loss is in the range of (0, *n*], the prediction is considered basically reliable and the model is not updated, whereas the model is upgraded when the value of loss is in the range of (*n*, 1).

## Experiments

### Environmental settings

TensorFlow 2.0 deep learning framework and Keras 2.3.1 high-level neural network API [42] are applied to build, train and save the proposed model, which will be further experimented under the PyCharm environment with the programming language of Python 3.7.

The hyperparameters considered are: the embedding dimension in word embedding, the number of hidden nodes in Bi-LSTM, the number of heads and output dimension in multi-attention, the minibatch size of pre-training and transfer learning, the number of epochs of pre-training and transfer learning, and the threshold of the loss function.

### Evaluation metrics

To validate the proposed model, Precision (*P*), Recall (*R*) and an evenly weighted F1-score (*F*1) are selected as evaluation metrics. *P* denotes the percentage of all predicted words whose true words were labelled by a human labeler, *R* denotes the percentage of all true words that were correctly predicted, and *F*1 indicates the overall performance as follows:

$$F1=\frac{2PR}{P+R}$$ (26)

In addition, words in QM-related texts are generally composed of restricted adjectives with two or more characters and a central word, so we define a concept of *long words* as words containing 5 or more words. And then we put forth a new metric of the *accuracy of long words* defined as the proportion of correctly predicted long words (denoted as *LT*) in all long words labelled by a human labeller (*LB*) as follows:

$$T=\frac{LT}{LB}$$ (27)

### Data preprocessing

#### Data set

We perform experiments on the following three datasets. 1) MSRA dataset. The dataset has 12.2M segments which is a standard used by the academic community to test CWS tools. 2) Enterprise QM standard. We collect 1 Enterprise QM standard ("Steel pipe flange (national standard series) Q/OMHJ003.2–2004") as a training dataset, which contains 231 sentences for training, about 3636 characters. 3) National QM standard. We collect 2 National QM standards of China (GB/T 13402–2010 Large Diameter Steel Pipe Flanges and GBT 9112–2010 Steel Pipe Flanges types and parameters) as a test dataset, which contains 507 sentences for test, about 9161 characters. A manual annotation process is followed to build the gold standard segmentation sequence on the enterprise QM standard dataset and national QM standard datasets.

#### Preprocessing

The datasets, such as “钢制管法兰/国家标准/系列” (Steel Pipe Flange/National Standard/Series), have to be converted into encodings before training. The specific steps are as following.

1. Labelling of texts. "BMES" scheme is used to label texts, where "B" represents the beginning of a word, "M" represents the middle of a word, "E" represents the ending of a word, and "S" represents a single word. Each character of texts is labelled according to the character’s position in words, which forms labelling sequences corresponding to the texts. The example text of “钢制管法兰/国家标准/系列” (Steel Pipe Flange/National Standard/Series) forms a sequence of "BMMMEBMMEBE".
2. Serialization of texts. Texts should be serialized to be learned by a model. A dictionary covering all characters is constructed by counting characters of texts, then texts are serialized by correspondences between text and related index in the dictionary. The example is converted to a sequence "[371,278,513,622,334,697,418,370,462,367,246]".
3. One-hot encoding of Labeling. One-hot encoding is the simplest word representation method, and it is enough to encode labelling. The four labelling in the "BMES" scheme are coded as follows: S {1,0,0,0}, B {0,1,0,0}, M {0,0,1,0}, E {0,0,0,1}. And the labeling sequences of example is transformed to a matrix.
4. Aligning of sequences. The length of each sentence may not be equal, which requires a standard length so that sentences longer than the standard length will be truncated, and sentences shorter than the standard length will be padded. Texts are padded with 0 and labeling are padded with -1.
5. Partition of datasets. The ratio of training and validation set is 8:2 on the MSRA and the enterprise QM standard datasets, whereas the national QM standard dataset is used as the test set.

### Experiment 1: Comparison between loss functions

The experiment is conducted to show the optimizing effect of the loss function by comparing the losses during training process before and after optimizing the loss function.

#### Pre-training

Hyperparameter settings are as follows: the embedding dimension is set to 100, the number of hidden nodes in Bi-LSTM is set to 200, the number of heads and output dimension are set to 4 and 200, respectively, the minibatch size is set to 256, and the number of epochs is set to 20. Then a pre-training part of the proposed model is trained with the MSRA dataset to get the weights of shared layers, including the embedding, the Bi-LSTM, the mutli-attention and the CRF.

#### Transfer learning with the CRF loss function

The shared layers are consistent with the hyperparameter settings in pre-training. The minibatch size is set to 4, and the number of epochs is set to 20. The adaption layer is set to a liner function without any hyperparameters, and the loss function is set to the loss function of CRF. The weights of shared layers are loaded to train the transfer learning part of the proposed model with the enterprise QM standard, and the changes of losses during the training process is recorded and plotted.

#### Transfer learning with the optimized loss function

The shared layers, the minibatch size, the number of epochs, and the adaption layer are consistent with the step of transfer learning with the CRF loss function. The loss function is changed to the optimized loss function of CRF, and the threshold is set to 0.1. The weights of shared layers are loaded to train the transfer learning part of the proposed model with the enterprise QM standard, and the changes of losses during the training process is recorded and plotted.

### Experiment 2: Comparison of performance among different methods

In the experiment, we compare the performance of the proposed model with several baseline methods. The baseline methods include: Jieba, the Bi-LSTM-CRF model [35], and the Bi-LSTM-CRF model with transfer learning.

#### Training of the Bi-LSTM-CRF model

The setting of hyperparameters is as follows: the embedding dimension is set to 100, the number of hidden nodes in Bi-LSTM is set to 200, the minibatch size is set to 256, and the number of epochs is set to 20. A mixed dataset is randomly shuffled composed of the MSRA and the enterprise QM standard. Then the Bi-LSTM-CRF model is trained by the mixed dataset.

#### Training of the Bi-LSTM-CRF model with transfer learning

A Bi-LSTM-CRF model which is consistent with the above hyperparameter settings is trained by the MSRA. Then the Bi-LSTM-CRF model with transfer learning shares the weights of the Bi-LSTM-CRF model, and is trained by the enterprise standard.

#### Training of the proposed model

According to the step of transfer learning with the optimized loss function in experiment 1, the proposed model is trained.

#### Evaluations of methods

The national QM standards are segmented by the above trained models and the Jieba. Then the segmentation results are compared with the labelling to evaluate the performance of methods from the three metrics of P, R, and F1.

### Experiment 3: Comparison of the accuracy of long words among different methods

In the experiment, we compare the accuracy of long words of the proposed model with the baseline methods in experiment 2. The baseline methods include: the Jieba, the Bi-LSTM-CRF model [35], and the Bi-LSTM-CRF model with transfer learning.

#### Predictions of methods

According to the step of training of the Bi-LSTM-CRF model, the Bi-LSTM-CRF model with transfer learning, and the proposed model in experiment 2, three trained models are achieved. The national QM standards are segmented by the trained models and the Jieba to get predictions.

#### Statistics of long words

Number of long words is counted separately in each prediction and the national QM standards.

#### Calculation of accuracy of long words

According to the Eq (27), the accuracy of long word of models is calculated from the statistics of long words.

### Experiment 4: Analysis of effects of word embedding dimensions and number of heads

In the experiment, we explore the effects of word embedding dimensions and number of heads.

#### Training of different word embedding dimensions in proposed model

Hyperparameters are set according to the setting in the step of training of the proposed model in experiment 2, except of word embedding dimensions. The dimensions used in the step is from 25 to 200, i.e., 25, 50, 75, 100, 125, 150, 175, 200. The proposed model is trained with different word embedding dimensions to get several trained proposed models.

#### Training of different number of heads in proposed model

Hyperparameters are set according to the setting in the step of training of the proposed model in experiment 2, except of number of heads. Due to the fact that a final output of a multi-head attention is a connection of outputs of different attention heads, the number of attention heads must be divisible by the dimension of the final output. So, different attention heads are selected to train the model, i.e., 0, 1, 2, 4, 5, 8, 10, 20. The proposed model is trained with different number of heads to get several trained proposed models.

#### Training of different word embedding dimensions in a Bi-LSTM-MA-CRF model

Hyperparameters are set according to the setting in the step of transfer learning with the CRF loss function in experiment 1, except of word embedding dimensions. Then the Bi-LSTM-MA-CRF model is trained by the enterprise QM standard with different word embedding dimensions to get several trained Bi-LSTM-MA-CRF models.

#### Evaluations of trained models on F1

The national QM standards are segmented by the above trained models, and F1 of each trained model is recorded and plotted.

## Results and discussion

### Comparison between loss functions

**Fig 4:**
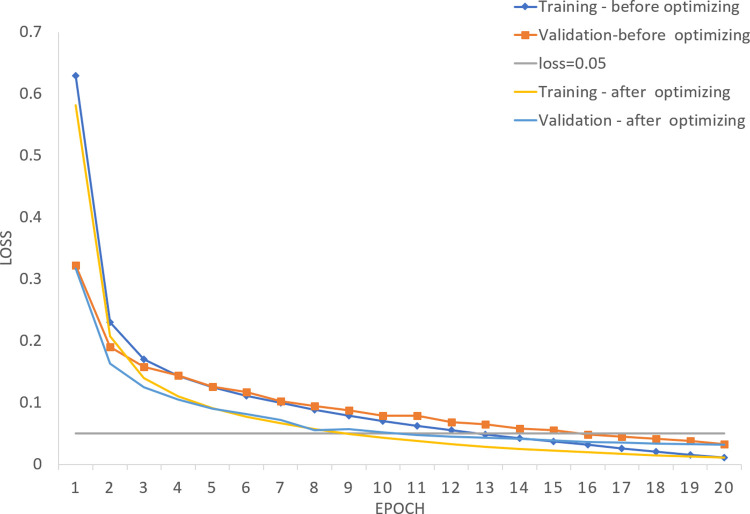
The losses of the training process before and after the optimization of loss function. A blue and an orange dot lines represent losses of training and validation set before optimization, whereas a yellow and a light blue lines represent losses of training and validation set after optimization. A gray line is the line when loss = 0.05.

Fig 4 shows losses before and after optimization. A blue and an orange dot lines represent losses of training and validation set before optimization, whereas a yellow and a light blue lines represent losses of training and validation set after optimization. A gray line is the line when loss = 0.05.

In Fig 4, losses of verification set reduce to 0.05 in the 8th round after optimization, whereas losses reduce to 0.05 until about the 14th round before optimization. Although the loss continued to decrease as the number of training epochs increases, the loss is just changed from 0.05 to 0.03 until the 20th round, with a decrease of only two percentage points, which is not significant improved. So, the model can stop learning when loss = 0.05 because of a good performance during training. Before and after optimization of the loss function, the losses of training and verification set decrease and gradually tends to 0 as the training epochs increase, that is, the model can achieve an ideal learning performance. It is shown that the losses of verification set are basically same between the models before and after optimization in the first and the last round, but the model after optimization only needs about 8 rounds of training to meet learning requirements, which is faster than the model before optimization. A possible cause is that the model is selective after optimization, which reduces a probability of adjusting to a local optimal direction when the loss is lower, improves a learning rate of the model to a global optimal direction, and accelerants a decline speed of loss function.

The learning rate of the model is improved by embedding a threshold, whereas the performance is unobvious. A possible reason is that no matter how "selective" the model is, the model always moves towards a global optimum, and is able to approach the optimum after lots of training. In Fig 4, the losses of verification set are basically consistent after 20 rounds of training before and after optimization, and the optimal effect is achieved.

### Comparison of performance among different methods

The metrics of P, R, and F1 of the proposed model are 97.3%, 98.3%, and 97.9%, respectively. Table 1 shows performances of the proposed model compared with the Jieba, the Bi-LSTM-CRF model and the Bi-LSTM-CRF model with transfer learning.

**Table 1:** Performances of methods.

Model	P	R	F1
Jieba	0.694	0.785	0.737
Bi-LSTM-CRF [35]	0.598	0.764	0.671
Bi-LSTM-CRF with transfer learning	0.936	0.955	0.946
mTL-Bi-LSTM-MA-CRF	0.973	0.983	0.979

Table 1 shows performances of models on test data. The performance of the encapsulated and generalized Jieba on QM-related texts is dissatisfactory, with metrics less than 80%, whereas a trained CWS model improves the metrics to more than 95%. As one of important preprocessing tasks in text mining, a better performance of CWS will support stronger for subsequent analysis tasks. Therefore, the proposed model with a better performance is significative in analysis of QM-related texts.

However, when a scale of corpus is small, the performance of a model with transfer learning is better than a model trained with mixed corpus. A possible reason is that the labeled QM-related texts is far less than the MSRA, and it is difficult to effectively learn the features from mixed corpus, even resulting in a negative effect. As a result, the performance of the Bi-LSTM-CRF model is slight lower than the Jieba. But transfer learning is different. The Bi-LSTM-CRF model with transfer learning applies knowledges of extracting feature learned from MSRA to QM-related texts. On this basis, learning transformation relationships between MSRA and QM-related texts, the model realizes a transfer of normal texts to QM-related texts, which greatly improves the performance of CWS.

Performances of the Bi-LSTM-CRF model with transfer learning and the mTL-Bi-LSTM-MA-CRF model on QM-related texts are fine, and each of metrics is more than 93%. But with the introduction of multi-attention mechanism, through an emphasis on key information and learning of long-distance relationships, the performance of the mTL-Bi-LSTM-MA-CRF model is further improved. Judging from the performance on test set, all metrices of mTL-Bi-LSTM-MA-CRF model are more than 97%, which is means that the proposed model may support subsequent analysis tasks more effectively.

### Comparison of accuracy of long words among different methods

The accuracy of long words of the proposed model is 98.4%. Table 2 shows the accuracy of long words of the proposed model compared with the Jieba, the Bi-LSTM-CRF model [35] and the Bi-LSTM-CRF model with transfer learning. Table 3 shows CWS examples of two sentences with long words segmented by the models.

**Table 2:** Accuracy of long words of methods.

Model	Total number of long words	Correct number of long words	Accuracy of long words
Jieba	184	5	0.027
Bi-LSTM-CRF [35]	184	19	0.103
Bi-LSTM-CRF with transfer learning	184	168	0.913
mTL-Bi-LSTM-MA-CRF	184	181	0.984

**Table 3:** Examples of CWS of methods.

Test texts	本/标准/代替/大直径碳钢管法兰/与/原/标准/相比/主要/ 变化/如下
	紧固件/六角头螺栓/和/六角螺母用沉孔
Jieba	本/标准/代替/大/直径/碳/钢管/法兰/与/原/标准/相比/主要/ 变化/如下
	紧固件/六角/头/螺栓/和/六角螺母/用/沉孔
Bi-LSTM-CRF [35]	本/标准/代替/大/直径/碳钢管法兰/与/原/标准/相比/主/要/变化/如下
	紧/固件/六角/头螺栓/和/六角螺母/用/沉孔
Bi-LSTM-CRF with transfer learning	本/标准/代替/大直径碳钢管法兰/与/原/标准/相比/主要/ 变化/如下
	紧/固件/六角头螺栓/和/六角螺/母/用/沉/孔
mTL-Bi-LSTM-MA-CRF	本/标准/代替/大直径碳钢管法兰/与/原/标准/相比/主要/ 变化/如下
	紧固件/六角头螺栓/和/六角螺母用沉孔

Comparing Table 2 with Table 1, the differences in the accuracy of long words between models are more obvious than performances. It may be resulted from that models are required to learn additional semantic rules for proper segmentation. For example, words such as "表面粗糙度" (surface roughness) are more likely to be combined by semantic rules in QM, while "表面" (surface) and "粗糙度" (roughness) are formed independently in normal texts. So, models need to learn more rules to segment correctly for long words. In test set, the accuracy of long words of the Jieba and the Bi-LSTM-CRF model are much lower than the overall performances. Therefore, long words are more likely to be mis-segmented in the Jieba and the Bi-LSTM-CRF model, and it is very important to alleviate the problem of excessing segmentation. The Bi-LSTM-CRF model with transfer learning is able to learn some special rules of semantic combination in QM-related texts. But due to influences of a Bi-LSTM, part of important information including special rules may be forgotten, so the accuracy of long words is slightly lower than overall performance. While combined with multi-attention mechanism, the proposed model got an accuracy of long words which is not lower than overall performance. Therefore, fusing multi-attention mechanism to strengthen learning of long-distance texts improves performance of CWS of QM-related texts.

Examples of "本/标准/代替/大直径碳钢管法兰/与/原/标准/相比/主要/ 变化/如下" (Compared with the original standard, this standard replaces the large diameter carbon steel pipe flange and the main changes are as follows.) and "紧固件/六角头螺栓/和/六角螺母用沉孔" (Fasteners hex head bolts and counterbore for hex nuts.) include three long words which are "大直径碳钢管法兰" (large diameter carbon steel pipe flange), "六角头螺栓" (fastener hex head bolts), and "六角螺母用沉孔" (counterbore for hex nut). Jieba and Bi-LSTM-CRF model segment the words excessively. The main reason is insufficient leaning of features in QM-related texts, resulting in unsatisfactory segmentations. The Bi-LSTM-CRF model after transfer learning and the mTL-Bi-LSTM-MA-CRF model are able to learn special rules through a training by small corpus. In addition, the Bi-LSTM-CRF model with transfer learning did not segment word "六角螺/母/用/沉/孔" (counterbore for hex nut) correctly in examples, and the segmentation result is far from the labelling result, which is means that the model still has a large room for improvement, and one of effective methods to improve is the multi-attention mechanism.

### Effects of word embedding dimensions and number of heads

**Fig 5:**
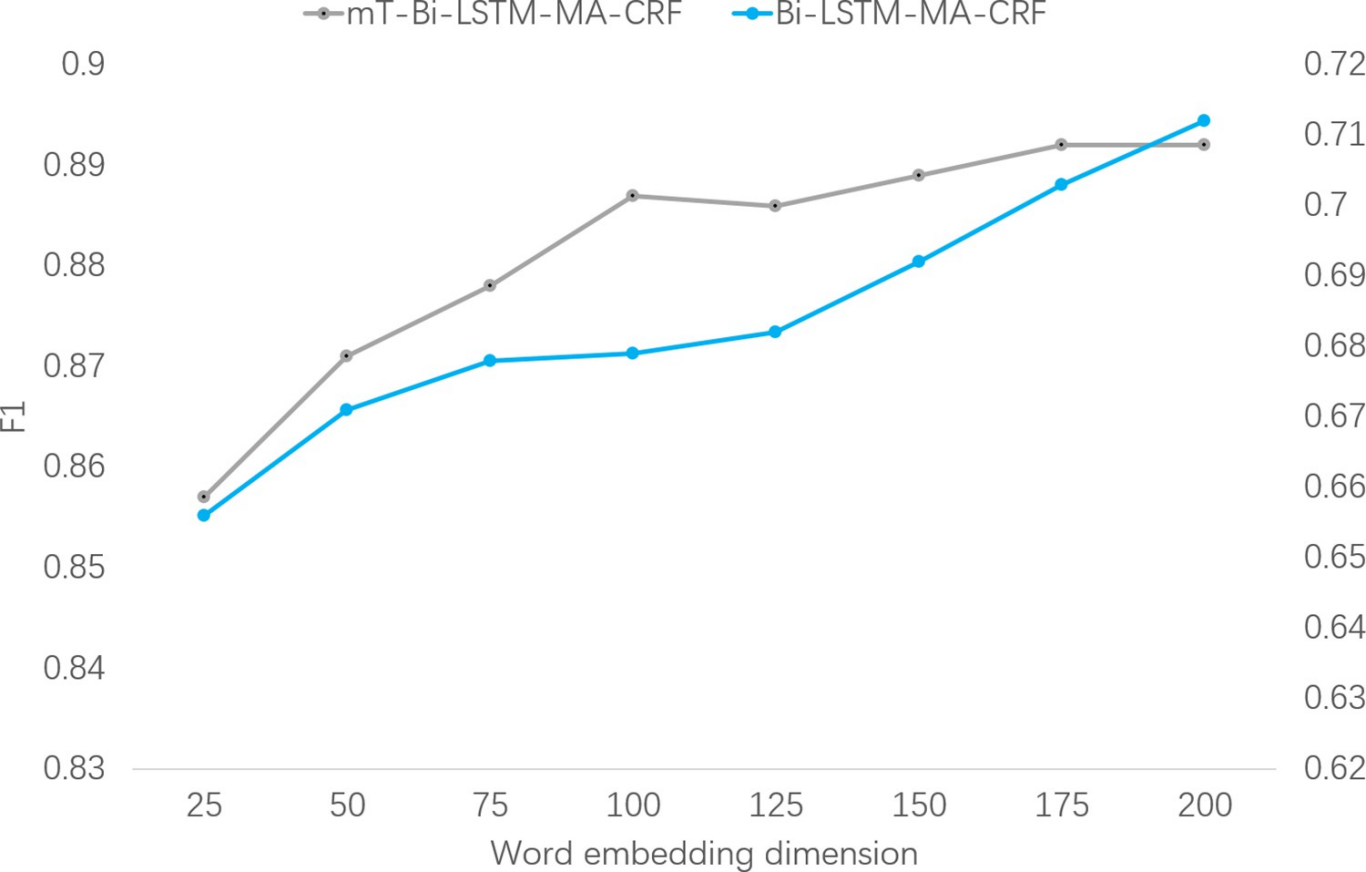
The F1 scores for different word embedding dimensions. A black dotted line and a blue dotted line represent the F1 score of the proposed model and the Bi-LSTM-MA-CRF model, respectively. The vertical axis of the black line is on the left, whereas the vertical axis of the blue line is on the right.

**Fig 6:**
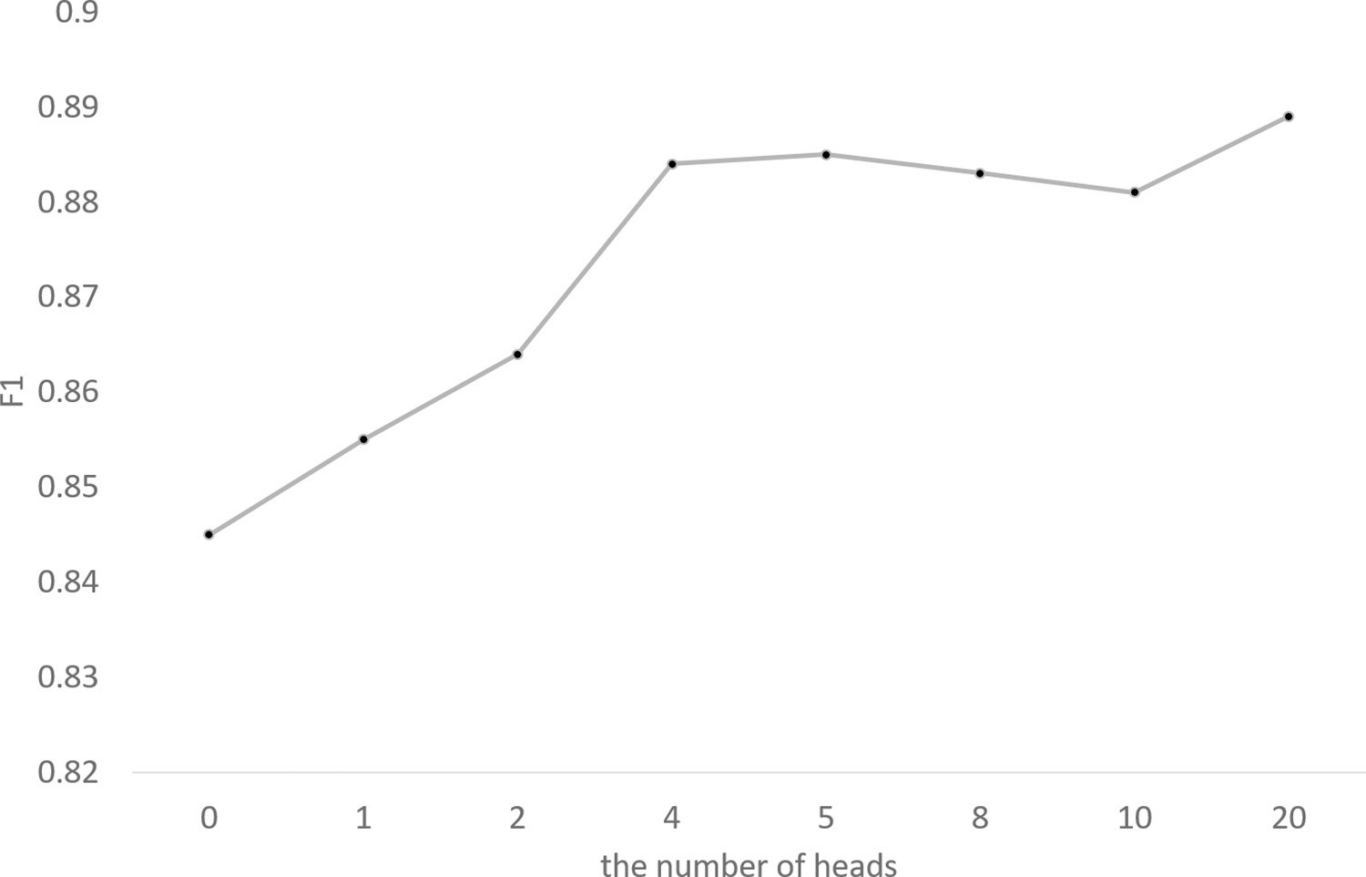
The F1 scores for different number of heads for multi-attention.

Fig 5 shows the F1 for different word embedding dimensions in validation set. A black dotted line and a blue dotted line represent the F1 of the proposed model and the Bi-LSTM-MA-CRF model, respectively. The vertical axis of the black line is on the left, whereas the vertical axis of the blue line is on the right. Fig 6 shows the F1 for different number of heads in validation set, where a head count of 0 indicates that the model has no attention layer at that time.

The F1 of the models increases as the word embedding dimension increases in Fig 5. The result of the experiment is in line with the fact that more embedding dimensions can represent richer word information, leading to a better learning effect of a model. In addition, the F1 of the model after transfer learning is significantly higher than the Bi-LSTM-MA-CRF model without pre-training weights, and the growth of scores is more steadily. This means that as the amount of text increases, the effect of word embedding dimension decreases. In the mT-Bi-LSTM-MA-CRF model, when the embedding dimension increases from 25 to 100, the F1 increase sharper than that from 100 to 200, whereas it is exactly the opposite in the Bi-LSTM-MA-CRF model. A possible explanation is that the mT-Bi-LSTM-MA-CRF model can already be able to express words clearly under a training with large-scale corpus, and the room for improvement by adding word feature dimensions is limited, whereas the training effect of small-scale corpus is limited, and more features are needed to express words in the Bi-LSTM-MA-CRF model. According to it, a pre-training enables the model to achieve better results in terms of word expression. Whether it is necessary to continually increase the dimension of word embeddings depends on relationships between the improvement of the performance and the increase of the calculation amount.

Fig 6 shows the F1 of the model increases with the increment of number of heads. Each head can be considered as a sampling. The result of the experiment is consistent with the fact that the performance will be better with more heads and more sampled viewing angles. In addition, increasing the number of heads can significantly improve the performance of the model when the number is small, and the improvement of the model becomes slow when the number increases to a considerable number by contrast. Therefore, a suitable number of heads is a better hyperparameter instead of a number which is as larger as possible.

## Conclusions

It is a trend to incorporate data analytics techniques into QM, and text analytics is one of most important methods. Domain-specific words generally contain important information and should be paid enough attention to, which requires an appropriate CWS method preserving full semantic information. By analyzing QM-related texts, six characteristics were summarized with territorial nature, grammatical combination rules, semantic combination rules, recurrence of characters, various structural paradigms, and incomplete expression, which motivates us to propose a new CWS model of mTL-Bi-LSTM-MA-CRF, integrating transfer learning, Bi-LSTM, Mutli-Attention and CRF. With the proposed model, the knowledge of word features extracted from general domain is transferred to the CWS for QM-related texts by shared layers and learning of transformational rule to support the segmentation of words obeying grammatical combination rules. Also, the activation function *tanh* of Bi-LSTM is replaced by *softsign* with the lower saturation, and the mutli-attention is introduced to meet the requirement of long-distance dependency. Furthermore, the loss function of CRF is optimized to reduce the effect of subtle differences between special and normal expressions, focusing on significant differences in larger distribution features. Four experiments are designed to analyze the improvement of the mTL-Bi-LSTM-MA-CRF model. The first comparison experiment between loss functions before and after an optimization process validated the improvement performance of the learning rate, as well the proposed model outperforms Jieba, the Bi-LSTM-CRF model, and the Bi-LSTM-CRF model with a transfer layer in the second comparison experiment of performance. And then the third comparison experiment of the accuracy of long words among the above four models showed that the proposed model could alleviate the excessive segmentation problem of long words with the introduction of multi-attention and transfer learning mechanisms. Besides, the fourth experiment analyzed the sensitivity of two hyperparameters, i.e., word embedding dimensions and number of heads, and recommended a range of pre-defined value for each one. Some limitations inevitably exist in this study, and there are several directions that could be explored in further research. Although the linear function to learn feature transformation rules provides a good result, the performance of transfer learning could be further improved by more accurately specifying a rule on transforming from the source domain to QM domain. In addition, the generalization ability and performance of the proposed model still need to be improved to adapt to more kinds of unstructured QM-related texts.

## Supporting information

S1 Dataset (ZIP)
